# Physical Limitations, Walkability, Perceived Environmental Facilitators and Physical Activity of Older Adults in Finland

**DOI:** 10.3390/ijerph14030333

**Published:** 2017-03-22

**Authors:** Erja Portegijs, Kirsi E. Keskinen, Li-Tang Tsai, Taina Rantanen, Merja Rantakokko

**Affiliations:** 1 Gerontology Research Center, Faculty of Sport and Health Sciences, University of Jyvaskyla, 40014 Jyväskylän yliopisto, Finland; kirsi.e.keskinen@jyu.fi (K.E.K.); ltsai@health.sdu.dk (L.-T.T.); taina.rantanen@jyu.fi (T.R.); merja.rantakokko@jyu.fi (M.R.); 2 Department of Sports Science and Clinical Biomechanics, University of Southern Denmark, 5230 Odense M, Denmark

**Keywords:** walking, physical function, walk-friendly environment, mobility limitation, age-friendly environment, physical activity, perceived environment, GIS, aging

## Abstract

The aim was to study objectively assessed walkability of the environment and participant perceived environmental facilitators for outdoor mobility as predictors of physical activity in older adults with and without physical limitations. 75–90-year-old adults living independently in Central Finland were interviewed (*n* = 839) and reassessed for self-reported physical activity one or two years later (*n* = 787). Lower-extremity physical limitations were defined as Short Physical Performance Battery score ≤9. Number of perceived environmental facilitators was calculated from a 16-item checklist. Walkability index (land use mix, street connectivity, population density) of the home environment was calculated from geographic information and categorized into tertiles. Accelerometer-based step counts were registered for one week (*n* = 174). Better walkability was associated with higher numbers of perceived environmental facilitators (*p* < 0.001) and higher physical activity (self-reported *p* = 0.021, step count *p* = 0.010). Especially among those with physical limitations, reporting more environmental facilitators was associated with higher odds for reporting at least moderate physical activity (*p* < 0.001), but not step counts. Perceived environmental facilitators only predicted self-reported physical activity at follow-up. To conclude, high walkability of the living environment provides opportunities for physical activity in old age, but among those with physical limitations especially, awareness of environmental facilitators may be needed to promote physical activity.

## 1. Introduction

Physical activity plays an important role in maintaining health and function in old age. Older adults are mostly physically active in the near vicinity of the home [1]. In accordance with the environmental press or person-environment fit model, an individual may limit his or her physical activity when the demands of the environment exceed the capabilities of the individual [2,3]. Low physical activity predisposes adults to loss of muscle strength, balance and endurance, which in turn may cause avoidance behavior and a vicious circle of declining physical activity and further declining capacity [4,5]. Lower physical capacity may lower older adults’ threshold to overcome environmental barriers [6,7], but attractive environments may still motivate older adults to go outdoors and be physically active. Currently, it is unclear whether attractive environmental features affect physical activity differently in older adults with and without physical limitations.

Geographical information such as land use mix, population density, and connectivity have been frequently combined into one walkability index reflecting possibilities to walk to different destinations [8]. A higher walkability index, indicating better walkability, has been associated with objectively assessed and self-reported physical activity, but not consistently [9,10]. Such objective environmental measures may not always correlate well with perceptions of the environment [11,12,13]. Subjectively perceived environmental factors reflect the capacity of the individual and the environment used by the individual [12]. Few studies have shown that reporting higher numbers of perceived environmental facilitators for outdoor mobility were associated with higher mobility function in older adults [14,15].

Person-environment relationships may be region-specific as environments and physical activity behaviors vary between countries [16,17,18]. Associations between walkability and physical activity have primarily been studied in cities in the USA, Australia and central Europe [9,17]. Urban areas in Nordic countries typically include more blue and green spaces. According to our knowledge, walkability was previously studied in one project among adults in Sweden [19]. Perceived environmental factors have been studied previously in Finland [14,15], but combined with objective environmental features only in one study on the immediate home environment [20]. Including both objectively assessed and subjective perceptions of the environment is important to provide a comprehensive picture of person-environment associations related to physical activity behavior in older adults.

Our aim was to study in older adults: (1) associations between objectively assessed walkability of the environment and participant perceived environmental facilitators for outdoor mobility, (2) their association with objective (step count) and self-reported physical activity at baseline, and (3) their association with self-reported physical activity over time. Based on the person-environment fit model, we hypothesized that associations between the environment and physical activity behavior in old age may differ by the presence or absence of physical limitations in the lower extremities; thus we stratified the analyses accordingly.

## 2. Materials and Methods

These cross-sectional and longitudinal analyses are part of the “Geographic characteristics, outdoor mobility and physical activity of older people” (GEOage) project. In this project, freely available geographic information characterizing the environment is linked to participant data of the “Life-space mobility in old age” (LISPE) cohort comprising 75–90-years-old community-dwelling people living in Jyväskylä and Muurame in Central Finland [21]. Briefly, a random sample of 2550 was drawn from the population register. Those living independently, able to communicate, residing in the recruitment area, and willing to participate were eligible to participate. In spring 2012, baseline data (*n* = 848; 62% female) were collected in a home interview [21]. One (*n* = 816; 62% female) and two (*n* = 761; 63% female) years later participants were re-interviewed over the phone [22]. At baseline, a subsample of 190 wore a tri-axial accelerometer for seven days following the baseline assessments. Participants were included based on the availability of accelerometers and willingness to participate. Valid days included ≥10 hours of accelerometer wear time. Technical problems (*n* = 4), <4 valid accelerometer days (*n* = 11) and >1 days in-between consecutive measurement days (*n* = 1) led to exclusion of data, thus leaving 174 participants (64% female) for the analyses [23]. Participants signed an informed consent prior to the data collection. The GEOage and LISPE study were approved by the University of Jyväskylä Ethical Committee.

### 2.1. Main Measures

*Lower extremity performance* was assessed with the Short Physical Performance Battery (SPPB), comprising of three tests that assess standing balance (narrow stance, semi-tandem stance, tandem stance), walking speed over 2.44 m, and five timed chair rises [24,25]. Each task was rated from 1 to 4 according to Finnish age- and gender-specific cut-off points [25]. Participants unable to complete the task due to balance- or mobility-related difficulties were assigned a score of 0, those unable to complete the task due to temporary medical conditions, wheel chair use, severe visual impairment, lack of a suitable chair or unwillingness to cooperate were not assigned a score (missing). By summing the task scores, a sum score (range 0–12) was calculated. For those with one task score missing, the final score was multiplied by 1.5. Higher scores indicate better performance. In order to stratify the sample for the analyses, participants were categorized according to the median; those with SPPB score ≤9 were labeled as having lower-extremity physical limitations.

*Self-reported physical activity* was assessed using a seven-point scale combining frequency and intensity of common physical activities [26]. Participants were categorized into at most light physical activity (at most light housework or gardening and short walks once or twice a week), moderate physical activity (at least moderate physical activity <3 h/week), and regular physical activity (moderate physical activity ≥4 h/week or strenuous physical activity). The self-reported physical activity scale and its categorization have shown to have concurrent validity with accelerometer assessed step counts and different measures of mobility [26].

In the substudy, average *daily step counts* were derived from an accelerometer (Hookie, tri-axial AM20 Activity Meter, Hookie Technologies Ltd., Espoo, Finland; sampling frequency 100 Hz and measurement range ±15 g_0_ (gravity of the Earth)) that was worn on the right hip for 7 days following the face-to-face interview at baseline [23]. Participants were instructed to wear the accelerometer daily from waking up to going to sleep, removing it for water activities only, and registered the wear times of the device in a diary.

The number of *perceived environmental facilitators* was calculated from the checklist for perceived environmental facilitators for outdoor mobility (PENFOM; 16 items), designed to identify the presence of environmental features that people perceive as facilitating their possibilities for outdoor mobility [15]. The PENFOM comprise parks, walking routes, nature, appealing landscape, familiar surroundings, good lighting, own yard, other people outdoors motivate, services or shops near, even sidewalks, walkways without steep hills, resting places by the walking route, peaceful and good quality pedestrian routes, no car traffic, no cyclist on walkways, and safe crossings.

Participants’ homes were located on the map by geocoding their home addresses in a geographic information system (GIS) [27]. The GIS-based *walkability index* was modified from Frank et al. [8]. In line with previous research [19], retail floor area ratio was omitted from the walkability index; thus leaving three components, land use mix, street connectivity, and population density, for which z-scores were calculated and summed to obtain the walkability index. Higher index scores indicate better walkability. The walkability index was calculated within 1 km from the participants’ home. Older adults frequently travel a 1 km distance on foot or by bike [28] and the respective distance has been used to define areas around participants homes for studying environmental factors [17,29].

*Land use mix* was based on the following land use types considered relevant for physical activity [30,31,32]: (1) Residential areas, (2) Services, (3) Sport and leisure facilities, and (4) Forest and semi-natural areas (build and natural green spaces) [33]; Land use mix represents the distribution of land use types in proportion to the dry land area within the 1 km circular buffer area around the participant’s home. We excluded water areas from the total surface area, which are abundantly present in the study area, but inaccessible and at the same time attract people to go outdoors [15]. Agricultural and industrial areas, which are not likely accessible or relevant for physical activity, were accounted for by including them in the total surface area [8]. Land use mix, ranging from 0 (homogeneity; single land use type) to 1 (heterogeneity; even distribution of land use types), was calculated as entropy value using Equation (1) [8]:

(1) $$\mathrm{Land}\;\mathrm{use}\;\mathrm{mix}=-\;\frac{\left(P1\right)\times \;\ln\left(P1\right)+\left(P2\right)\times \ln\left(P2\right)+\left(P3\right)\times \ln\left(P3\right)+\left(P4\right)\times \ln\left(P4\right)}{\ln\left(4\right)}\;P=\frac{\mathrm{surface}\;\mathrm{area}\;\mathrm{of}\;\mathrm{respective}\;\mathrm{land}\;\mathrm{use}\;\mathrm{type}\;}{\mathrm{total}\;\mathrm{surface}\;\mathrm{area}\;\mathrm{of}\;\mathrm{buffer}\;\mathrm{without}\;\mathrm{water}\;\mathrm{areas}}\;\ln=\mathrm{natural}\mathrm{logarithm}$$

*Street connectivity* was the number of street intersections of walkable ways in all possible directions along the road network up to 1 km distance from the home. Only at least 3-way intersections were included [8] and street intersections within 10 m from each other were merged for the calculations. To obtain walkable ways, motorways, trails, winter roads, railroads and ferry lines over water were omitted from the road network [34]. Absolute resident numbers in the 1 km × 1 km squares of the study area [35] where participants resided was used as an indicator of *population density* [11].

### 2.2. Confounding Variables

*Age, sex* and *time lived in the current home,* calculated based on the date of the latest address change (missing values were imputed with the average (*n* = 25)), were derived from the national register. The number of self-reported *chronic conditions* were calculated from a list of 22 physician diagnosed chronic diseases and an additional open-ended question about any other physician diagnosed chronic conditions. For those participating in the accelerometer substudy, *daily wear time of the accelerometer* was calculated from the self-report diary data. To retain as many participants in the analyses as possible, missing accelerometer wear time values were imputed with the average of the respective individual (if missing 1 or 2 days; *n* = 15) or the group (if missing for all days; *n* = 1) after visual inspection of the data to assure ≥10 h of wear time [23]. The assessment month (January–June; 1–6) was used to account for *climatological circumstances.*

### 2.3. Statistical Analyses

Nine participants were excluded from all analyses due to two or three missing task scores of the lower extremity performance test. Thus, baseline analyses included 839 participants and analyses on step counts 174 participants. Longitudinal analyses were conducted excluding participants who moved prior to any study follow-ups (*n* = 18), and those without any follow-up assessment of self-reported physical activity (*n* = 34). Consequently, 787 participants were included in the longitudinal analyses and self-reported physical activity was based on the latest available assessment. All analyses were conducted in two ways; including all eligible participants (non-stratified) and stratified by lower-extremity physical limitations (SPPB score 0–9 versus 10–12).

Z-scores of the walkability index were divided into tertiles. The number of perceived environmental facilitators was used as such as a dependent variable or divided into tertiles when used as an independent variable. Group differences were tested with independent *T*-tests (continuous variables) and Chi-square tests (categorical variables). Spearman correlation coefficients were calculated between objective and perceived environmental variables. In all analyses, we consistently found similar associations for “moderate” and “regular” self-reported physical activity when compared to at most light self-reported physical activity. Consequently, we merged these two categories into one category “at least moderate physical activity”. 

Walkability index and perceived environmental facilitators were included in separate regression models. All regression analyses were first adjusted for age and sex, and then in addition adjusted for number of chronic conditions, years lived in the current home, years of education, and climatological circumstances one at a time and jointly (fully adjusted). In the non-stratified models, lower-extremity physical limitations was added as an independent variable. Regression coefficients (B and standard error) derived from *Generalized Linear Models* (GLM) are reported to describe cross-sectional associations between walkability variables and number of perceived environmental facilitators (identity link) at baseline (study aim 1). In addition, GLM were used to study associations between walkability variables and step counts (log link transformation) at baseline (study aim 2). Models studying step counts were additionally adjusted for accelerometer wear time. *Logistic regression models* were used to calculate odds ratios (OR and 95% confidence intervals) for reporting at least moderate self-reported physical activity at baseline (study aim 2). Logistic regression analyses stratified by baseline physical activity were conducted to predict at least moderate self-reported physical activity at the latest follow-up (study aim 3).

SPSS Statistics 22 (IBM Inc., Armonk, NY, USA) was used for all statistical analyses, and statistical significance was set at *p* < 0.05. ArcMap 10.3.1 (ESRI, Redlands, CA, USA) was used to join map layers and create GIS variables.

## 3. Results

In the full baseline sample, participants with lower-extremity physical limitations were older, and they had more chronic conditions, lower levels of education and lower levels of physical activity than those without physical limitations (Table 1). In the accelerometer subsample, those with lower-extremity physical limitations had lower levels of physical activity and a higher number of chronic conditions. The Spearman correlation coefficient between the walkability index and perceived environmental facilitators was 0.2 (*p* < 0.001).

### 3.1. Cross-Sectional Associations

#### 3.1.1. Perceived Environmental Facilitators

Living in an area characterized by a higher walkability index was associated with reporting higher numbers of environmental facilitators (Table 2). Each tertile of higher walkability progressively increased the number of environmental facilitators reported by participants with lower-extremity physical limitations, while among those without physical limitations the regression coefficients were similar for the middle and highest tertile of walkability.

#### 3.1.2. Physical Activity

Table 3 shows that participants living in areas with the highest walkability index had higher step counts than those living in an area with the lowest walkability index. However, in participants with lower-extremity physical limitations, the association was statistically significant only in the fully adjusted model (*p* = 0.019). Perceived environmental facilitators were not associated with step counts irrespective of lower-extremity physical limitations.

The odds for reporting at least moderate physical activity at baseline were higher for participants perceiving higher numbers of environmental facilitators than for those in the lowest tertile (Table 4), and the association was especially strong among those with lower-extremity physical limitations. Living in an area with the highest walkability index was associated with higher odds for reporting at least moderate physical activity, but statistical significance was reached in the non-stratified sample only.

### 3.2. Longitudinal Associations

Walkability index was not associated with self-reported physical activity at the latest follow-up (Table 5). In *participants with at least moderate physical activity at baseline*, higher numbers of perceived environmental facilitators for outdoor mobility were associated with reduced odds for maintaining at least moderate self-reported physical activity over time in those with lower-extremity physical limitations only, but the association attenuated after full adjustment (*p* = 0.070). In *participants with low physical activity at baseline*, however, reporting the highest number of perceived environmental facilitators more than doubled the odds for reporting at least moderate physical activity at the latest follow-up, but the associations reached statistical significance in the non-stratified sample only.

## 4. Discussion

This study provides a comprehensive picture of associations between objectively assessed walkability of the environment and perceived environmental facilitators for outdoor mobility, and how these factors relate to objective and subjective measures of physical activity in older adults with and without lower-extremity physical limitations. The results show that higher numbers of perceived environmental facilitators and better walkability were associated with higher physical activity levels, but not consistently across physical activity measures and time points. Furthermore, the current results show that better walkability was associated with higher numbers of perceived environmental facilitators, although rather weakly. Finally, the presence of lower-extremity physical limitations affected the strength of some person-environment relationships.

A major part of older adults’ physical activity is carried out in the home environment [1], but moving through larger areas may add to the accumulation of physical activity [36]. The area, where an individual with physical limitations moves, shrinks and ultimately becomes restricted to the neighborhood [37,38]. Consequently, people with physical limitations may consider a smaller area when reporting perceived facilitators for outdoor mobility than those without physical limitations who move through larger areas in their everyday life [28]. In addition, those without physical limitations may be more motivated and better able to reach attractive destinations for outdoor mobility and physical activity beyond their own neighborhood. This may explain why the associations between perceived environmental facilitators and self-reported physical activity were somewhat weaker for participants without lower-extremity physical limitations.

The walkability index is an objectively assessed measure characterizing the environment. In line with previous research, a higher walkability index did not fully translate into higher numbers of environmental factors facilitating outdoor mobility reported by older adults [11,12]. Theoretically, more road intersections and more people living in a designated area implies better availability of or access to services such as shops [8,39]. Having services within walking distance from home has been found to motivate older adults to go outdoors and be physically active [39,40]. However, other factors such as nature and water areas may also motivate people to go outdoors and be physically active [15,30]. The land use mix component of walkability to some extent covers such facilitators, but it does not capture specific preferences of individuals [32]. Because of the larger effort required to move outdoors and be physically active in those with physical limitations [41], these individuals are more likely to convey strategies to conserve their energy [38,42]. Consequently they may be more specific about which features in the neighborhood environment they consider to be attractive. The walkability index is a rather generic measure [43]. In future research, it may be useful to focus on more detailed environmental features including availability, accessibility and use of specific destinations for physical activity such as services or natural areas [30,39,40].

The relatively weak association between walkability assessed based on map data and perceived environmental facilitators for outdoor mobility may be related to the measurement dimension. Perceived environmental facilitators reflect the environment that an individual uses, the ability and preferences of the individual [12,13]. The question on perceived environmental facilitators used in this study asked specifically about the presence of environmental features that motivate individuals to go outdoors in their home environment [15]. Information on use of such environments was not directly available, but use is likely. Objectively assessed GIS-based variables, such as the walkability index, do not take into account individual preferences or abilities of the individual, and, in addition, require assumptions to be made regarding neighborhood boundaries. Previous research showed that definitions of neighborhood boundaries and the area where an individual actually moves (activity-spaces) vary in size and orientation between individuals [29,44]. Individuals may demonstrate preferences for a certain direction due to attractive routes and destinations or to avoid difficult terrain or heavy traffic. Consequently, the area used to define the neighborhood in the GIS-based walkability index may not be an accurate reflection of the environment used by the individual [28,29,44]. By incorporating behavioral patterns in area definitions reflecting actual use of the environment, e.g., by using GPS or interactive mapping tools [29,45], it may be possible to assess more accurately walkability with objective GIS-based measures.

This is one of the first studies looking at longitudinal associations between objective and perceived environmental variables and self-reported physical activity. In the current study, walkability was not associated with maintenance of or increase in self-reported physical activity over the two-year follow-up. Previously, proximity of popular destinations was associated with maintenance of physical activity [40], and higher walkability was associated with smaller declines in walking for transport, but not leisure walking over time in older adults [31]. In addition, it has been observed that adults changed their physical activity behavior in response to relocation and subsequential changes in destinations [46]. In all, earlier studies point toward highly walkable environments having a positive, even though modest, influence on maintaining physical activity at a higher level. The lack of association in the current study may be partly explained by the high age of the participants in the current study, predisposing them to a decline in health and functioning and, consequently, also a decline in physical activity.

The longitudinal findings of perceived environmental facilitators and physical activity were equivocal. Higher numbers of perceived environmental facilitators were associated with an increased likelihood to become at least moderately physically active over the follow-up, but at the same time, also with reduced likelihood of maintaining at least moderate self-reported physical activity for those with lower-extremity physical limitations. The finding that perceived environmental facilitators increased the likelihood of becoming at least moderately physically active over the follow-up may be real or an artifact due to temporary restriction of habitual physical activity at baseline, as awareness of environmental facilitators may indicate larger exposure to the environment [13]. Either way, this stresses the importance of older adults’ awareness and recognition of environmental facilitators for outdoor mobility in their home environment as a means to overcome restrictions in physical activities.

The finding that perceived environmental facilitators reduced the likelihood of maintaining higher levels of physical activity was surprising. Previous research showed higher numbers of perceived environmental facilitators decreasing the risk for development of walking difficulty [14]. It is possible that those perceiving few environmental facilitators for outdoor mobility at baseline (the reference group), may not move outdoors as frequently, but they may be more adapted to the situation (e.g., by using compensatory strategies) [13,42]. Consequently, they may be better able to maintain their physical activity over time than those with lower-extremity physical limitations who reported more environmental facilitators. However, it is also possible that over time participants modified their perceptions of the environment due to declining physical function and subsequent changes in goals and valuation of meaningful activities [42,47]. Unfortunately, we could not take this into account in the analyses due to lacking data on perceived environmental facilitators at the follow-ups.

Even in longitudinal studies, it is difficult to disentangle whether the environment affects physical activity or whether the environment was chosen partly because of favorable characteristics for walking [46,48]. In our study, the average duration that an individual had lived in the same home was well over 20 years. In the past decades the city of Jyväskylä has experienced a marked increase in population and infrastructure. It is likely that both the environment and the preferences of individuals may have changed and thus the effect of neighborhood selection reduced over time. However, when physical function starts to decline, older adults may accommodate by moving to an environment better suited to their needs [49]. These are likely areas with better access to services [49], which likely coincides with better walkability. The current data suggest that participants with lower-extremity physical limitations living in the least walkable areas had lived on average 5 years longer in the current home than those living in more walkable areas. As a result, for participants with physical limitations only, adjustment for the duration lived in the current home markedly strengthened the association between the walkability index and step counts. 

Strengths of this study include a population-based sample with large sample size and few missing values. This is one of few studies assessing environmental features both objectively and as perceived by the individual, and physical activity behavior of older adults. Lower-extremity physical limitations were assessed objectively. The walkability measures were categorized, in line with previous studies [8,44], which may have reduced variability, potentially leading to underestimation of associations. On the other hand, a continuous measure of the walkability index may not capture variation relevant for physical activity [44], especially considering non-linearity of certain associations. Furthermore, the walkability index has been widely used, but not standardized. There is no consensus about calculation strategies of its components [17]. GIS data were available for the calculation of a walkability index in Finland, although not fully in accordance with the originally proposed variables by Frank et al. [8]. Furthermore, both self-reported and accelerometer-based physical activity measures may be prone to bias [50]. Accelerometers may underestimate physical activity especially among those walking slowly (in the current study those with lower-extremity physical limitations) and those engaging in low-impact physical activities (e.g., cycling, swimming or skiing) [50]. Finally, the analyses using objectively assessed step counts were based on a relatively small sample size, warranting caution when interpreting the results.

## 5. Conclusions

Considering the trend of aging in community homes and the preventive effect of physical activity on maintaining health and function into old age, knowledge of attractive environmental features motivating older adults with physical limitations in the lower extremities to go outdoors is essential. This study provides a comprehensive picture of associations between objectively assessed walkability, perceived environmental facilitators and physical activity in community-dwelling older adults in Finland. Findings suggest that walk-friendly environmental design may provide opportunities for physical activity in old age. Associations between perceived environmental facilitators and physical activity suggest that creating awareness of attractive environmental factors in the home environment may be needed to promote physical activity, especially when physical function starts to decline. However, also motivation may play a role in this respect. Intervention studies are needed to confirm reported associations.

## Figures and Tables

**Table 1:** Participant and environmental characteristics in the full baseline sample (*n* = 839) and the physical activity subsample (*n* = 174).

Characteristic	Full Baseline Sample			Physical Activity Subsample		
	With Physical Limitations (*n* = 310)	Without Physical Limitations (*n* = 529)		With Physical Limitations (*n* = 44)	Without Physical Limitations (*n* = 130)	
	Mean ± SD	Mean ± SD	*p*	Mean ± SD	Mean ± SD	*p*
Age (year)	81.3 ± 4.2	80.2 ± 4.2	<0.001 ^a^	81.1 ± 4.4	80.1 ± 4.2	0.202 ^a^
Time lived in home (year)	22.4 ± 14.9	23.3 ± 14.4	0.383 ^a^	24.7 ± 15.0	24.3 ± 14.5	0.879 ^a^
Chronic diseases (*n*)	5.1 ± 2.5	3.9 ± 2.3	<0.001 ^a^	5.0 ± 2.6	4.0 ± 2.1	0.025 ^a^
Education (year)	8.8 ± 4.0	10.1 ± 4.2	<0.001 ^a^	9.7 ± 4.3	9.7 ± 3.9	0.995 ^a^
Step count (*n*)	-	-	-	1762.7 ± 2109.4	3185.9 ± 2840.2	0.001 ^a^
Perceived environmental facilitators (*n*)	6.1 ± 3.8	6.2 ± 3.5	0.473 ^a^	6.5 ± 3.6	6.0 ± 3.3	0.336
Sex (% Female)	63.9	61.1	0.461 ^b^	68.2	62.3	0.604 ^b^
Assessment month (% January–April)	68.7	67.9	0.859 ^b^	63.6	58.8	0.339 ^b^
Self-reported physical activity	-	-	<0.001 ^b^	-	-	0.009 ^b^
(% At most light)	54.8	24.2		38.6	16.9	
(% Moderate)	26.8	32.1	-	36.4	42.3	-
(% Regular)	18.4	43.7	-	25.0	40.8	-
Walkability	-	-	0.335 ^b^	-	-	0.620 ^b^
(Lowest)	30.0	35.0		36.4	44.6	
(Middle)	35.5	32.7	-	34.1	28.5	-
(Highest)	34.5	32.3	-	29.5	26.9	-

^a^ Independent *t*-test, **^b^** Chi-square test.

**Table 2:** Regression coefficients for reporting higher numbers of perceived environmental facilitators according to tertiles of the walkability index (*n* = 848).

Variable	Tertile	Non-Stratified						With Physical Limitations						Without Physical Limitations					
		Crude ^a,c^			Fully Adjusted ^b,c^			Crude ^a^			Fully Adjusted ^b^			Crude ^a^			Fully Adjusted ^b^		
		B	SE	*p*	B	SE	*p*	B	SE	*p*	B	SE	*p*	B	SE	*p*	B	SE	*p*
Walkability index	Lowest	0.0	-	-	0.0	-	-	0.0	-	-	0.0	-	-	0.0	-	-	0.0	-	-
	Middle	1.6	0.3	<0.001	1.4	0.3	<0.001	1.3	0.5	0.010	1.2	0.5	0.028	1.8	0.4	<0.001	1.5	0.4	<0.001
	Highest	1.9	0.3	<0.001	1.5	0.3	<0.001	2.3	0.5	<0.001	1.8	0.6	0.001	1.8	0.4	<0.001	1.4	0.4	<0.001

B = Regression coefficient, SE = Standard error. Generalized linear models adjusted for ^a^ age and sex, ^b^ age, sex, number of chronic conditions, years lived in the home, years of education, and climatologic circumstances, and ^c^ additionally for lower-extremity physical limitations.

**Table 3:** Regression coefficients for higher step counts according to tertiles of perceived environmental facilitators or tertiles of the walkability index (*n* = 174).

Variable	Tertile	Non-Stratified						With Physical Limitations						Without Physical Limitations					
		Crude ^a,c^			Fully Adjusted ^b,c^			Crude ^a^			Fully Adjusted ^b^			Crude ^a^			Fully Adjusted ^b^		
		B	SE	*p*	B	SE	*p*	B	SE	*p*	B	SE	*p*	B	SE	*p*	B	SE	*p*
Perceived facilitators	Lowest	0.0 ^^^	-	-	0.0	-	-	0.0	-	-	0.0	-	-	0.0	-	-	0.0	-	-
	Middle	-	-	-	0.2	0.2	0.391	0.2	0.5	0.688	0.3	0.5	0.546	0.4	0.2	0.086	0.3	0.2	0.154
	Highest	-	-	-	0.2	0.2	0.283	0.0	0.5	0.955	0.3	0.6	0.626	0.3	0.2	0.148	0.3	0.2	0.268
Walkability index	Lowest	0.0	-	-	0.0 ^~^	-	-	0.0	-	-	0.0	-	-	0.0	-	-	0.0	-	-
	Middle	0.0	0.2	0.881	0.0	0.2	0.832	−0.2	0.4	0.649	0.1	0.4	0.761	0.1	0.2	0.652	0.1	0.2	0.617
	Highest	0.5	0.2	0.004	0.5	0.2	0.010	0.3	0.5	0.476	1.1	0.5	0.019	0.5	0.2	0.014	0.5	0.2	0.045

B = Regression coefficient, SE = Standard error. Generalized linear models adjusted for ^a^ age, sex, and accelerometer wear time, ^b^ age, sex, accelerometer wear time, number of chronic conditions, years lived in the home, years of education, and climatologic circumstances, and ^c^ additionally for lower-extremity physical limitations. ^^^ Model could not be computed as the Hessian matrix is singular. ^~^ The maximum number of step-halvings was reached but the log-likelihood value cannot be further improved.

**Table 4:** The odds for reporting at least moderate physical activity at baseline according to tertiles of perceived environmental facilitators or tertiles of the walkability index (*n* = 848).

Tertile	Non-Stratified				With Physical Limitations				Without Physical Limitations			
	Crude ^a,c^		Fully Adjusted ^b,c^		Crude ^a^		Fully Adjusted ^b^		Crude ^a^		Fully Adjusted ^b^	
	OR	95% CI	OR	95% CI	OR	95% CI	OR	95% CI	OR	95% CI	OR	95% CI
Perceived facilitators												
Lowest	1.0	-	1.0	-	1.0	-	1.0	-	1.0	-	1.0	-
Middle	**2.6**	**1.8–3.9**	**2.8**	**1.9–4.2**	**4.6**	**2.4–9.0**	**4.9**	**2.5–9.9**	**1.9**	**1.2–3.2**	**2.1**	**1.2–3.6**
Highest	**3.5**	**2.3–5.3**	**3.5**	**2.3–5.4**	**5.9**	**3.0–11.7**	**6.4**	**3.1–13.1**	**2.6**	**1.5–4.5**	**2.6**	**1.5–4.6**
Walkability index												
Lowest	1.0	-	1.0	-	1.0	-	1.0	-	1.0	-	1.0	-
Middle	1.1	0.8–1.6	1.1	0.8–1.6	0.8	0.4–1.4	0.8	0.5–1.6	1.5	0.9–2.4	1.5	0.9–2.5
Highest	**1.6**	**1.1–2.3**	**1.6**	**1.1–2.5**	1.4	0.8–2.6	1.6	0.8–3.1	1.7	1.0–2.8	1.6	0.9–2.7

Bold values indicate *p* < 0.050, OR = Odds ratio, 95% CI = 95% confidence interval. Logistic regression models adjusted for ^a^ age and sex, ^b^ age, sex, number of chronic conditions, years lived in the home, years of education, and climatologic circumstances, and ^c^ additionally for lower-extremity physical limitations.

**Table 5:** The odds for reporting at least moderate physical activity (PA) by the latest follow-up according to tertiles of perceived environmental facilitators or tertiles of the walkability index stratified by physical activity level at baseline (BL).

Tertile	Non-Stratified				With Physical Limitations				Without Physical Limitations			
	Crude ^a,c^		Fully Adjusted ^b,c^		Crude ^a^		Fully Adjusted ^b^		Crude ^a^		Fully Adjusted ^b^	
	OR	95% CI	OR	95% CI	OR	95% CI	OR	95% CI	OR	95% CI	OR	95% CI
*Moderate PA at BL*												
Perceived facilitators												
Lowest	1.0	-	1.0	-	1.0	-	1.0	-	1.0	-	1.0	-
Middle	0.6	0.3–1.4	0.6	0.2–1.3	0.3	0.1–1.3	0.3	0.0–1.4	0.8	0.3–2.2	0.8	0.3–2.2
Highest	0.5	0.2–1.1	0.5	0.2–1.1	**0.2**	**0.0–0.9**	0.2	0.0–1.0	0.8	0.3–2.1	0.7	0.3–1.9
Walkability index												
Lowest	1.0	-	1.0	-	1.0	-	1.0	-	1.0	-	1.0	-
Middle	1.1	0.6–2.0	1.1	0.6–2.2	2.0	0.7–6.0	2.6	0.8–8.0	0.7	0.3–1.6	0.7	0.3–1.8
Highest	1.2	0.6–2.2	1.1	0.6–2.3	1.0	0.4–2.3	1.3	0.5–3.5	1.6	0.6–3.9	1.3	0.5–3.4
*Low PA at BL*												
Perceived facilitators												
Lowest	1.0	-	1.0	-	1.0	-	1.0	-	1.0	-	1.0	-
Middle	1.1	0.6–2.0	1.1	0.6–2.2	0.6	0.2–1.8	0.6	0.2–1.8	1.5	0.6–3.7	1.7	0.7–4.4
Highest	**2.4**	**1.1–4.9**	**2.5**	**1.2–5.2**	2.3	0.9–5.8	2.5	0.9–6.9	2.4	0.8–6.8	2.6	0.9–7.6
Walkability index												
Lowest	1.0	-	1.0	-	1.0	-	1.0	-	1.0	-	1.0	-
Middle	1.1	0.6–2.2	1.0	0.5–2.1	1.2	0.4–3.0	1.3	0.5–3.6	1.1	0.4–2.9	0.9	0.3–2.4
Highest	0.7	0.3–1.3	0.6	0.3–1.3	0.6	0.2–1.9	0.7	0.2–2.4	0.7	0.3–1.7	0.6	0.2–1.6

Bold values indicate *p* < 0.050, OR = Odds ratio, 95% CI = 95% confidence interval. Logistic regression models adjusted for ^a^ age and sex, ^b^ age, sex, number of chronic conditions, years lived in the home, years of education, and climatologic circumstances, and ^c^ additionally for lower-extremity physical limitations.
